# *Clostridium butyricum* in combination with specific immunotherapy converts antigen-specific B cells to regulatory B cells in asthmatic patients

**DOI:** 10.1038/srep20481

**Published:** 2016-02-09

**Authors:** Hong-Ying Liao, Li Tao, Jian Zhao, Jie Qin, Gu-Cheng Zeng, Song-Wang Cai, Yun Li, Jian Zhang, Hui-Guo Chen

**Affiliations:** 1 Department of Thoracic Surgery, Caner Center of Guangzhou Medical University, Guangzhou, 510092, China; 2 Department of Neonate Laboratory, Guangzhou Woman & Children’s Medical Center, Guangzhou, 510623, China; 3 Department of Radiology, Third Affiliated Hospital, SUN Yat-sen University, Guangzhou 510630, China; 4 Department of Microbiology, Zhongshan School of Medicine, Key Laboratory for Tropical Diseases Control of the Ministry of Education, Sun Yat-sen University, Guangzhou, 510080, China

## Abstract

The effect of antigen specific immunotherapy (SIT) on asthma is supposed to be improved. Published data indicate that administration of probiotics alleviates allergic diseases. B cells play important roles in the pathogenesis of allergic diseases. This study aims to modulate antigen specific B cell property by the administration of *Clostridium butyrate* (*CB*) in combination with SIT. The results showed that after a 3-month treatment, the total asthma clinical score and serum specific IgE were improved in the patients treated with SIT, which was further improved in those treated with both SIT and *CB*, but not in those treated with *CB* alone. Treatment with SIT and *CB* increased p300 and STAT3 activation, up regulated the IL-10 gene transcription and increased the frequency of peripheral antigen specific B cells. In conclusion, administration with SIT in combination with *CB* converts Der p 1 specific B cells to regulatory B cells in asthma patients allergic to Der p 1. The data suggest a potential therapeutic remedy in the treatment of allergic diseases.

Allergic asthma is an airway disease mediated by antigen specific IgE. The prevalence of allergic asthma is increasing worldwide in the recent decades^1^. The pathogenesis of asthma has not been fully appreciated yet. Current understanding about the pathogenesis of asthma includes that overproduction of allergen specific IgE; the IgE binds the high affinity receptor of IgE on the surface of mast cells to make mast cells sensitized. Re-exposure to specific allergens activate the sensitized mast cells and trigger the mast cells to release allergic mediators to evoke clinical allergic symptoms^2^. Although research in this area advanced rapidly in recent years, the treatment of asthma is still unsatisfactory^3^. Therefore, to invent novel therapeutic remedies for asthma is of great significance.

The antigen specific immunotherapy (SIT) is the only available effective treatment to target the allergic diseases, such as asthma, instead of the symptoms^4^. SIT is to introduce small doses of the specific antigens to the patients via subcutaneous injection or sublingual absorption, including a build-up phase and a maintenance phase. In the build-up phase, increasing doses of allergens are introduced to patients weekly, while in the maintenance phase, a fixed dose of allergen is introduced to patients monthly^4^,^5^. One of the mechanisms of SIT is to induce antigen specific immune tolerance in the body, including inducing regulatory T cells (Treg) and regulatory B cells (Breg)^6^. The transforming growth factor-β (TGF-β) and interleukin (IL)-10 are the most common cytokines released from the immune regulatory cells^6^. These mediators suppress other immune effector cell activities so as to suppress the allergic symptoms. To date, the mechanism of immune regulatory cells has not been fully appreciated yet.

Probiotics are “live microorganisms which, when administered in adequate amounts, confer a health benefit on the host”, as described by the World Health Organization. Probiotics are normal microbial flora in the intestine to facilitate fermenting ingested food products, secrete lactic acid and are associated with immune regulation^7^. Probiotics should meet the following requirements: Reduction or exclusion of pathogenic adherence in the intestine; production of acids, H_2_O_2_, and producing bacteriocin against pathogens; short chain fatty acids production; biosynthesis of Vitamin K; fermentation of indigestible dietary fiber; positive influence on peristalsis; safety, noninvasiveness, noncarcinogenicity, and co-aggregation mechanisms to form a normal balanced gut microbiota^8^,^9^. It is pointed out that administration of probiotics has a recognizable effect on allergic dermatitis, but less effective for airway allergies^10^. Thus, we hypothesize that probiotics may facilitate SIT to regain immune tolerance in the airway mucosa of patients with airway allergies. In this study, we treated allergic asthma patients with both SIT and one strain of probiotics, the CB. The results showed that the addition of CB dramatically enhanced the therapeutic effect on asthma via inducing the antigen specific Bregs.

## Results

### *CB* enhances the therapeutic effect of SIT on asthma

Published data indicate that probiotics improved immunity in the body^11^. SIT is a therapeutic remedy using in the treatment of allergic diseases; yet the therapeutic efficacy is to be improved. We inferred that combination of SIT and probiotics might enhance the therapeutic effect on asthma than either SIT or using probiotics alone. To test the hypothesis, we treated mite-sensitized asthma patients with SIT in combination with or without *Clostridium Butyricum* (CB). The asthma parameters were measured before and 3 months after the treatment. Table 1 displays the asthma symptom score and serum specific IgE levels before SIT and 3 months after. The results showed that treatment with SIT reduced the total asthma symptoms and the serum specific IgE levels, which was markedly improved by the treatment with SIT/CB, but was not apparently improved in those treated with CB alone. The results indicate that administration with CB enforces the effect of SIT on asthma.

### CB promotes generation of specific regulatory B cells by SIT

**Figure 1:**
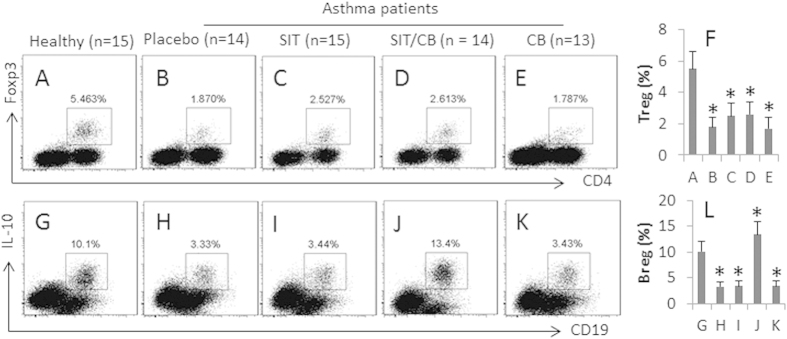
*C. butyricum* (CB) and SIT generate IL-10^+^ B cells in asthma patients. PBMCs were collected from each asthma patient after treating with SIT or/and CB for 3 months. The cells were analyzed by flow cytometry. The dot plots show the frequency of Treg (**A–F**) and Breg (**G–L**). The bars (mean ± SD) are the summarized data of the dot plots. *p < 0.05, compared with the healthy group.

One of the scientific foundations of SIT for allergic disease is to induce immune regulatory cells^6^. To see if CB promotes the generation of immune regulatory cells by SIT, we collected the peripheral blood from the asthma patients before SIT and 3 months after. The peripheral blood mononuclear cells (PBMC) were analyzed by flow cytometer. The results showed that the frequency of CD4^+^ Foxp3^+^ Treg was 3.46% in healthy subjects (Fig. 1A), which was significantly lower in patients treated with placebo (1.87%; Fig. 1B,F). Treatment with SIT only slightly increased Tregs (2.52%; Fig. 1C,F), which was further increased a little after treating with both SIT and CB (2.61%; Fig. 1D,F). Treatment with CB alone did not improve the Treg generation (1.79%; Fig. 1E,F). The results suggest that treatment with SIT or/and CB only marginally increases Treg generation in asthma patients.

The IL-10^+^CD19^+^ regulatory B cells (Breg) are also an important fraction of the immune regulatory cells in the body. We also assessed the Bregs in the PBMCs of asthma patients. The results showed that the frequency of Breg was lower in asthma patients treated with placebo than that in healthy controls (Fig. 1G,H,L). Treating with SIT alone did not increase Bregs apparently (Fig. 1I,L), which were much increased by the treatment with both SIT and CB (Fig. 1J,L), but not with CB alone (Fig. 1K,L). The results imply that treatment with SIT/CB can induce Breg development in asthma patients.

### Combination of SIT and CB modulates antigen specific B cell properties

**Figure 2:**
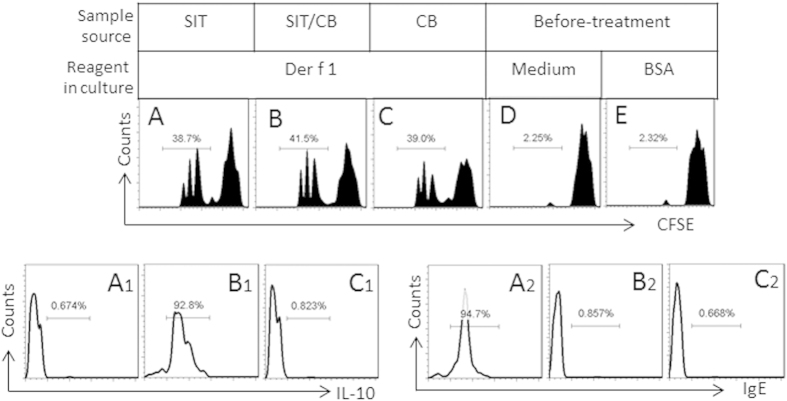
CB and specific antigens modulate specific B cell properties. (**A–E**) CD19^+^ B cells were isolated from PBMCs of asthma patients before or after treatment with SIT or/and CB for 3 months. The cells were labeled with CFSE, cultured in the presence of Der p 1 (1 μg/ml; or medium alone, or BSA as an irrelevant antigen) and anti-CD40 (20 ng/ml) for 3 days. The gated histograms indicate the frequency of proliferating B cells. (**A1–C1**) the histograms indicate the frequency of IL-10^+^ B cells in the gated cells of (**A–C**). (**A2–C2**) the histograms indicate the frequency of IgE^+^ cells in the gated cells of (**A–C**). Each parameter was averaged from the results of samples collected from three patients; the samples from individual patients were processed separately.

We next analyzed the antigen-specific B cells from asthma patients. CD19^+^ B cells were isolated from PBMCs of asthma patients treated with SIT or/and CB for 3 months, and analyzed by flow cytometry. The results showed that, after incubated with the specific antigen in the culture, about 38.7%, 41.5% and 39.0% B cells proliferated in the samples from patients treated with SIT, SIT/CB and CB respectively (Fig. 2A–C). Exposure to medium alone or BSA (an irrelevant antigen) did not induce proliferation of the B cells (Fig. 2D,E). Further analysis showed about 92.8% proliferated cells were also IL-10 positive in the cells from patients treated with SIT/CB, which did not occur in those treated with neither SIT alone or CB alone (Fig. 2A–C1). On the other hand, the proliferated B cells of the samples from patients treated with SIT alone showed 94.7% IgE positive cells, which did not occur in those from patients treated with SIT/CB or CB alone (Fig. 2A2–C2). The results suggest that treatment of asthma patients with SIT/CB induces IL-10^+^ B cells while treatment with either SIT alone induces IgE positive cells, and treated with CB alone does not induce neither IL-10^+^ B cells nor IgE positive cells.

### Treatment with Der p 1 and CB suppresses IgE expression in B cells

**Figure 3:**
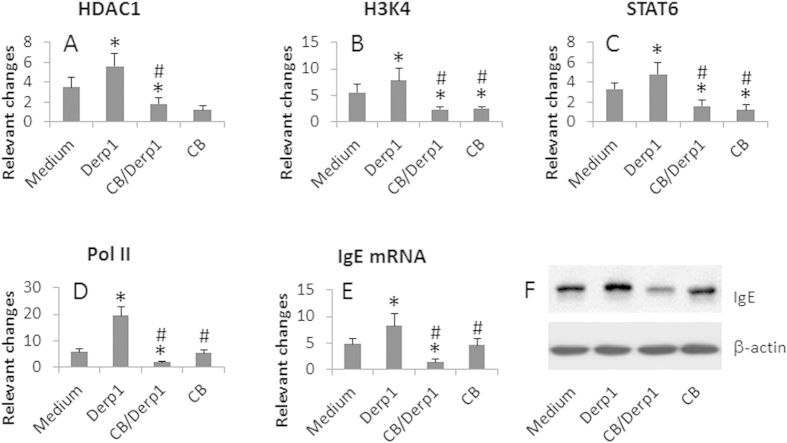
Administration with CB/Der p 1 modulates IgE expression in DerBC. Der p 1 specific B cells (DerBC) were isolated from mite-allergic asthma patients with the Derp1-tetramer. The treatment was denoted in the figure. (**A–D**), the cells were analyzed by ChIP assay. The bars indicate the relevant changes of HDAC1 (**A**), H3K4 (**B**), STAT6 (**C**) and RNA polymerase II (Pol II) (**D**). (**E,F**) the cell extracts were analyzed by RT-qPCR and Western blotting. (**E**) the bars indicate the IgE mRNA. (**F**) the blots indicate the IgE protein. Data of bars are presented as mean ± SD. *p < 0.01, compared with the medium group. ^#^p < 0.01, compared with the Derp1/CB group. The data are representatives of 3 independent experiments. The full length gel graphs are presented in the supplemental materials.

We harvested Der p 1-specific B cells (DerBC) from the patients with the Derp1-tetramer. The B cells were analyzed by ChIP assay. As shown by Fig. 3A–D, higher levels of HDAC1, H3K4, STAT6 and RNA polymerase II were observed at the IgE promoter locus in patients treated with Der p 1 vaccine alone, which were markedly suppressed in patients treated with both Der p 1 vaccine and CB. In those treated with CB alone, however, although the levels of HDAC1 and H3K4 were suppressed, the levels of STAT6 and RNA polymerase II were not altered. The expression of IgE in the DerBCs was similarly altered to the changes at the IgE promoter locus after the treatment (Fig. 3E,F). The results indicate that treatment with Der p 1 alone increases the expression of IgE in the DerBCs, which can be suppressed by the treatment with both Der p 1 and CB. Treatment with CB alone can suppress the HDAC1 and H3K4 at the IgE promoter locus, but cannot suppress the levels of STAT6 and RNA polymerase.

### Blocking HDAC1 by butyrate facilitates IL-10 expression in antigen specific B cells

**Figure 4:**
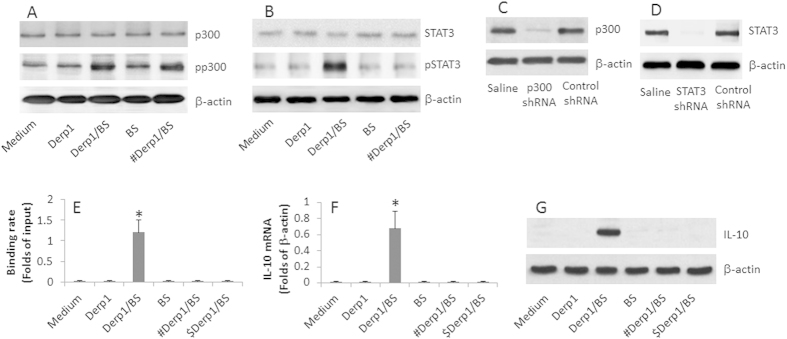
SIT and butyrate induce IL-10 expression in antigen specific B cells. DerBCs were isolated from PBMCs of asthma patients without specific treatment. The cells were treated as denoted on the figures. (**A,B**) the Western blots show the phosphorylation of p300 (**A**) and STAT3 (**B**). (**C,D**), the blots show the RNAi results of p300 (**C**, #) and STAT3 (**D**, $). (**E,F**) the bars indicate the binding rate of IL-10 gene by pSTAT3 (**E**) and the mRNA levels of IL-10 (**F**) in the DerBCs. (**G**), the Western blots show the protein levels of IL-10 in the DerBCs. The data are representatives of 3 independent experiments. The full length gel graphs are presented in the supplemental materials. # = deficiency of p300. $ = deficiency of STAT3.

The data of Fig. 1 show that administration with CB facilitates SIT to induce IL-10 expression in B cells. We next took further insight into the mechanism of the IL-10 expression. Previous studies demonstrate that p300 and STAT3 are associated with the expression of IL-10^12^. We then evaluated the activation of p300 and STAT3 in DerBCs after exposure to the specific antigen, Der p 1, or/and butyrate in the culture. As shown by Fig. 4, exposure to Der p 1 did not induce recognizable phosphorylation of p300 and STAT3, which was markedly up regulated in the presence of both Der p 1 and butyrate sodium while exposure to butyrate sodium alone did not alter the levels of pp300 and pSTAT3 in the DerBCs. Knockdown of p300 abolished the phosphorylation of STAT3 while knockdown of STAT3 did not affect the pp300 levels, indicating STAT3 phosphorylation is the downstream of p300 phosphorylation. We also found that the pSTAT3 highly bound to the IL10 gene promoter, and up regulated the expression of IL-10, but not IgE. The results indicate that activation of p300 and STAT3 play a critical role in the specific antigen/butyrate-induced IL-10 production of the DerBCs.

## Discussion

The treatment of allergic asthma is refractory. SIT is the only specific remedy for asthma treatment currently. The therapeutic efficacy of SIT is unsatisfactory. The present data show that the addition of CB significantly increased the therapeutic efficacy of SIT. Administration with both SIT and CB markedly reduced the total asthma symptom score and suppressed the serum antigen specific IgE levels. Data from the mechanistic experiments showed that treatment with SIT alone only somewhat increased Bregs while treatment with both SIT and CB significantly increased the frequency of antigen specific Bregs in the peripheral blood, but not in those treated with either SIT alone or CB alone.

The World Health Organization proposes that SIT is the only specific remedy currently for the treatment of allergic diseases. Various SIT efficacies have been recorded. Kaufman reported an 81% improvement of SIT in atopic dermatitis after a 2-year subcutaneous treatment^13^. Warner reported that asthma patients treated with SIT showed the greatest improvement in symptoms^14^. On the other hand, Glover indicated that the efficacy of SIT on asthma is “uncertain”^15^ while Galli even observed no difference between SIT group and control group^16^. Our results of using SIT alone are somewhat similar to the results of Glover^15^; although some positive results were observed, it was not satisfactory. The results suggest some improvement of SIT may be required.

The ability to improve immunity by probiotics has been identified. Some investigators have applied probiotics in the treatment of allergic diseases. Probiotic administration can alter the components of local microflora, and can modulate the Toll-like receptors in the gut, leading to the activation of dendritic cells and a Th1 response; the latter can thus suppress the Th2 response in allergic diseases^17^. Probiotics also stimulate B cells to secrete mucosal IgA as well as allergen-specific B cell responses^18^. Our study has uncovered a novel aspect of probiotics. By working together with SIT, CB-derived butyrate promotes IL-10 gene transcription and IL-10 production in the antigen specific B cells. It is recognized that IL-10-producing B cells have immune suppressor functions^19^. We also observed that the generated IL-10-producing B cells inhibited effector B cell proliferation (data not shown). Our data suggest that the combination of SIT with CB may dramatically enforce the therapeutic efficacy of SIT.

HDAC1 is one of the factors in regulating gene transcription. The regulatory effect can be negative or positive depending on the micro environment. Our data demonstrate that, after exposure to specific antigens *in vitro*, high levels of HDAC1 phosphorylation were detected in DerBCs, which was associated with IgE production in the DerBCs. It is observed that the presence of butyrate abolished the Der p 1-induced IgE production in DerBCs. The CB we used in the present study can secrete butyrate. Since IgE is the main mediator evoking allergic reactions, the combination of SIT and CB has a great significance to be applied in the treatment of allergic diseases.

IL-10 is one of the immune regulatory cytokines^19^. Our data show that the combination of SIT and CB increased the production of IL-10 by the DerBCs, indicating a generation of Bregs. Lee reported that the IL-10-producing B cells were important in the inhibiting the non-IgE-mediated food allergy^20^. Our data reveal another aspect of this event; the IL-10-producing B cells were also induced in the patients treated with SIT and CB. STAT3 and p300 are vital components in the signal transduction pathway of IL-10 production^12^. Our data are in line with the previous reports; we observed that upon exposure to specific antigens and butyrate in the culture, levels of pp300 and pSTAT3 were up regulated markedly.

In summary, the data show that the combination of SIT with CB has a better therapeutic efficacy in the inhibition of asthma than using either SIT alone or CB alone. The underlying mechanism is that this strategy inhibits IgE production and promotes IL-10 production in the antigen specific B cells. The data suggest a potential therapeutic remedy in the treatment of allergic diseases.

## Materials and Methods

### Reagents

The antibodies of HDAC1, pSTAT6, p300, pp300, STAT3, pSTAT3, H3K4ac, RNA polymerase II, shRNA kits of p300 and STAT3 were from Santa Cruz Biotech (Santa Cruz, CA). The fluorochrome-labeled antibodies of Foxp3, CD4, IL-10, CD19 and IgE were from BD Biosciences (Franklin Lakes, NJ). The biotinylated IgE antibody was from Abcam (Cambridge, MA). Magnetic cell sorting kits were from Miltenyi Biotech (San Diego, CA). The house dust mite vaccine was from Wowu Biotech (Hangzhou, China). *Clostridium butyricum* was from Shenzhen Kexing Biotech (Shenzhen, China). The Der p 1 protein was from Dr. Zhijiang Liu (Shenzhen University, China). PCI-32765 was purchased from Chem Blink (Shanghai, China). Reagents for real time RT-PCR and Western blotting were from Invitrogen (Carlsbad, CA). Protein G, ChIP kit and butyrate sodium were from Sigma Aldrich (St. Louis., MO).

### Patients

Asthma patients with mild to moderate clinical symptoms, solely sensitized to mite allergen were recruited to this study. During the period of observation, patients were required not taking extra treatment (this is the reason to select patients with mild symptoms). If extra treatment besides SIT was required, the patients were excluded from the study and switched to proper treatments. The sensitization was diagnosed through the skin prick test (Alutard SQ, ALK-Abelló, Denmark), specific IgE (UniCAP®, Phadia, Sweden) and the history of asthma by the physicians of our department. Patients were randomized to 4 groups treated with SIT, SIT/CB, CB and placebo respectively. The study was approved by the Human Ethic Committee at Sun Yat-sen University. A written informed consent was obtained from each patient (or the patients’ representatives, if the age was under 12). The study was carried out in accordance with the approved guidelines.

### Treatment

The SIT was administered with an extract of *Dermatophagoides pteronyssinus* (Der p) extract absorbed to aluminium hydroxide (Alutard SQ, ALK-Abelló, Denmark) in accordance with our established protocol (1 μg/ml; wk1: 0.1 ml; wk2: 0.2 ml; wk3, 0.3 ml; wk4: 0.4 ml; and 0.5 ml biweekly from wk5 to wk13) via subcutaneous injection. The patients remained in the clinic under observation for 1 h after the injection.

A group of patients was treated with both SIT and oral CB. The protocol of SIT was the same as described above. In addition, the patients took two capsules of CB (420 mg/capsule; Kexing Biotech, Shangdong, China) twice daily.

The CB group patients took two capsules of CB (420 mg/capsule; Kexing Biotech, Shangdong, China) twice daily. The placebo group patients were treated with saline (to replace Der p vaccine) and capsules containing vehicle.

### Total asthma symptom score (TAS)

The TAS was recorded semi-quantitatively by patients before SIT and 3 months after. 0: No symptoms; 1: Mild; 2: Moderate; 3: Severe.

### Assessment of serum Der p-specific IgE antibody

The blood was obtained from the elbow vein before SIT and 3 months after. The serum Der p-specific IgE level was measured by enzyme-linked immunosorbent assay (ELISA). The micro-plates were coated with mite crude extracts (10 mg/ml) and incubated at 4 °C overnight. The plates were blocked with 5% fetal bovine serum (FBS) in PBS for 30 min at room temperature. After washing with PBS containing 0.05% Tween 20 (PBST), the serum samples (1/20 dilution) were added to the micro-plates and incubated overnight at 4 °C. After washing with PBST, the plates were incubated with anti-human IgE biotinylated mAb for 1 h at room temperature. After washing with PBST, the plates were incubated with horseradish-peroxidase (HRP) streptavidin conjugate at room temperature for 30 min. The wells were washed and then incubated with TMB substrate (Sigma Aldrich) for 30 minutes at room temperature. The enzyme reaction was stopped by adding 2 N H_2_SO_4_. The plates were read with a microplate reader at 450 nm.

### Immune cell isolation

The peripheral blood mononuclear cells (PBMC) were isolated from the peripheral blood by density gradient centrifugation. Immune cells were then isolated from the PBMCs using magnetic cell sorting (MACS) kits following the manufacturer’s instructions. The cell purity was greater than 96% as checked by flow cytometry. Isolated immune cells were cultured with RPMI1640 medium supplemented with 10% FBS, 100 U/ml penicillin, 0.1 mg/ml streptomycin and 2 mM L-glutamine. In the case of B cell culture, anti-CD40 (20 ng/ml) was added to the culture. The cell viability was checked by Trypan blue exclusion assay.

### Construction of a Der p-specific Tetramer

To isolate the Der p 1 specific B cells (DerBC), a tetramer was constructed following reported procedures^21^,^22^ with a minor modification. The biotinylated Der p 1 was incubated with magnetic particle-conjugated streptavidin for 30 min at room temperature. Unconjugated reagents less than 10 kDa were filtered through a filter tube by centrifugation. The Der p 1 tetramers were collected for DerBC isolation.

### DerBC Isolation

Following published procedures^22^, PBMCs were isolated from the peripheral blood, and Der p 1 tetramer was added to the cells at a concentration of 2 μg/ml and incubated for 30 min at room temperature. The cells were then passed through the columns in the magnetic apparatus provided by Miltenyi Biotech. Cells were collected, washed with acidic phosphate-buffered saline (PBS) (pH 3) to remove the bound Der p 1 on the cell surface, and transferred to RPMI 1640 medium for further experiments.

### Flow cytometry

In the surface staining, the cells were stained with fluorochrome-labeled primary antibodies (0.5 μg/ml) for 1 h at room temperature. If necessary, the cells were stained intracellularly by fixing and permeabilizing, and followed by incubating with fluorochrome-labeled antibodies (0.5 μg/ml), or an isotype IgG (used as a control) for 1 h. In the case of assessing the antigen specific B cell proliferation, the isolated antigen specific B cells were isolated by the Der p specific tetramer and labeled with CFSE, and cultured in the presence of specific antigens for 3 days. After washing with PBS, the cells were analyzed by flow cytometry. The data were analyzed by software Flowjo. The data of isotype IgG staining were used as a gating reference.

### Western blotting

The total proteins were extracted from cells. The proteins were fractioned by SDS-PAGE and transferred onto a PVDF membrane. The membrane was blocked by incubating with 5% skim milk for 30 min, incubated with the primary antibodies (100 ng/ml) overnight at 4 °C, and followed by incubating with the second antibodies (conjugated with peroxidase) for 1 h at room temperature. Washing with TBST (Tris buffered saline Tween 20) was performed after incubation. The membrane was developed by the ECL. The results were photographed with an Image Station (KODAK Image Station 4000Pro).

### Real time RT-PCR (RT-qPCR)

The total RNA was extracted from the cells with the TRIzol reagent. The cDNA was synthesized using a reverse transcription kit. The qPCR was carried out in a real time PCR device (CFX96 Touch™ Real-Time PCR Detection System; Bio Rad) with the SYBR Green Master Mix. The primers using in the present study include: IgE, forward, tagtgactctgatgccaccc; reverse, ccccagaggtccaagtaaca. IL-10, forward, ggcgctgtcatcgatttctt; reverse, atagagtcgccaccctgatg. The results were calculated with the 2^−∆∆Ct^ method and presented as a relative value against the internal control β-actin.

### Chromatin IP (ChIP)

The cells were fixed with 1% formaldehyde to cross link the DNA binding protein. The samples were sonicated to shear DNA along with bound proteins into small fragments, and incubated with antibodies of interest and protein G overnight at 4 °C to bind antibodies specific to the DNA-binding protein to isolate the complex by precipitation. After cross-link-reversal and DNA purification, qPCR was performed on the samples and inputs. The primers of the promoter regions include: Ig heavy chain germline Igϵ (tgggcctgagagagaagaga and agctctgcctcagtgctttc) and IL-10 (cctcctatccagcctccatg and tgtacaccatctccagcaca). The results are presented as relevant value against the input.

### RNA interference (RNAi)

The gene of p300 and STAT3 was knockdown by RNAi with commercial shRNA kits following the manufacturer’s instructions. The knockdown effect was assessed by Western blotting.

### Statistics

The data are presented as mean ± SD. The difference between two groups was determined with the Student t test or ANOVA if more than two groups. A p < 0.05 was set as a significant criterion.

## Additional Information

**How to cite this article**: Liao, H.-Y. *et al.* *Clostridium butyricum* in combination with specific immunotherapy converts antigen-specific B cells to regulatory B cells in asthmatic patients. *Sci. Rep.* **6**, 20481; doi: 10.1038/srep20481 (2016).

## Supplementary Material

Supplementary Information
