# Supplementary material for: Clostridium butyricum in combination with specific immunotherapy converts antigen-specific B cells to regulatory B cells in asthmatic patients
Source: Sci Rep. 2016 Feb 9;6:20481. doi: 10.1038/srep20481 (PMC4746628; doi:10.1038/srep20481)
Supplement: Supplementary Information [file srep20481-s1.pdf]

Supplemental materials

***Clostridium butyricum* in combination with specific immunotherapy converts antigen-specific B cells to regulatory B cells in asthmatic patients**

Running title: Immunotherapy converts regulatory B cells

Hong-Ying Liao[1#], Li Tao[2#]， Jian Zhao[3], Jie Qin[4]， Gu-Cheng Zeng[5], Song-Wang Cai[1], Yun Li[1], Jian Zhang[1], Hui-Guo Chen[1]

1. Department of Thoracic surgery, Clinical research center of Chest Tumor, Third Affiliated Hospital, SUN Yat-sen University, Guangzhou 510630, China
2. Department of Neonate Laboratory, Guangzhou Woman & Children's Medical Center, Guangzhou, 510623, China
3. Department of Thoracic Surgery, Caner of Guangzhou Medical University, Guangzhou, 510092, China
4. Department of radiology, Third Affiliated Hospital, SUN Yat-sen University, Guangzhou 510630, China
5. Department of Microbiology, Zhongshan School of Medicine, Key Laboratory for Tropical Diseases Control of the Ministry of Education, Sun Yat-sen University, Guangzhou, 510080, China

Corresponding author: Dr. Hong-Ying Liao, Department of Thoracic surgery, Clinical research center of Chest Tumor, Third Affiliated Hospital, SUN Yat-sen University, Guangzhou 510630, China. Tel: +86-20-85252246. Email: hongyingrliao@outlook.com

#These authors contributed equally to this work

Full length gel graphs (requested to present by the journal)

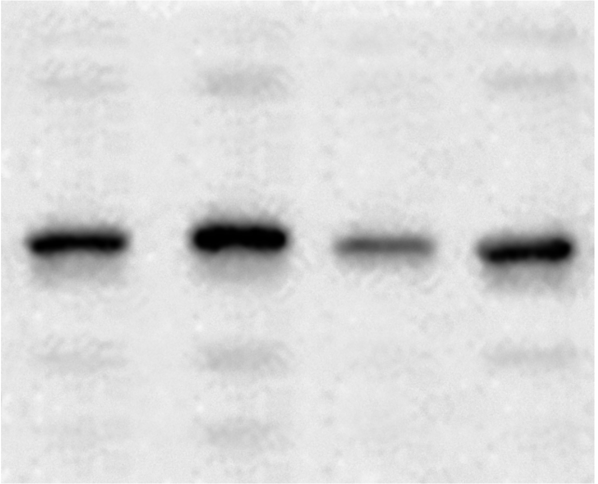

Fig.3F. Ige

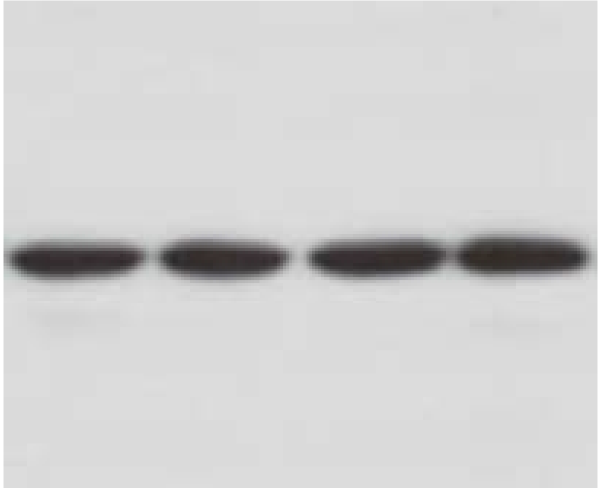

Fig.3F. Beta-actin

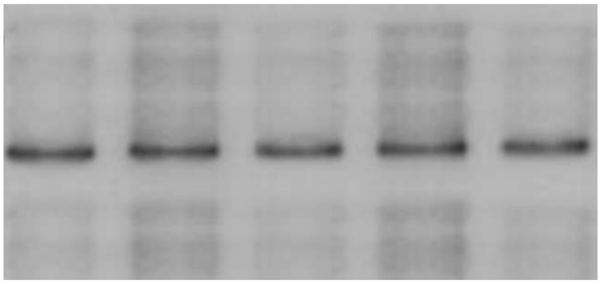

Fig.4A. p300

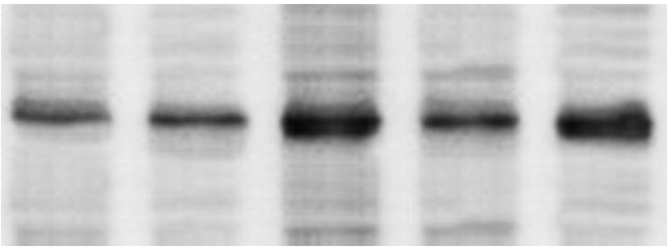

Fig.4A.pp300

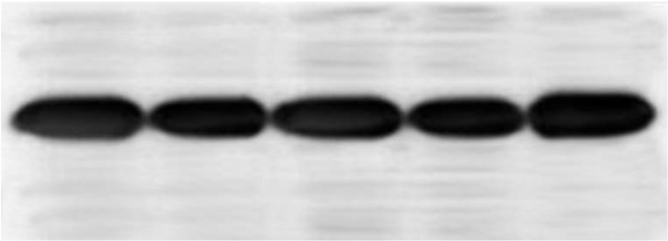

Fig.4A. Actin

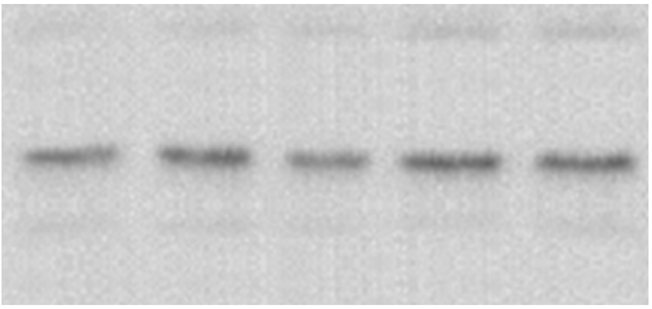

Fig.4B. STAT3

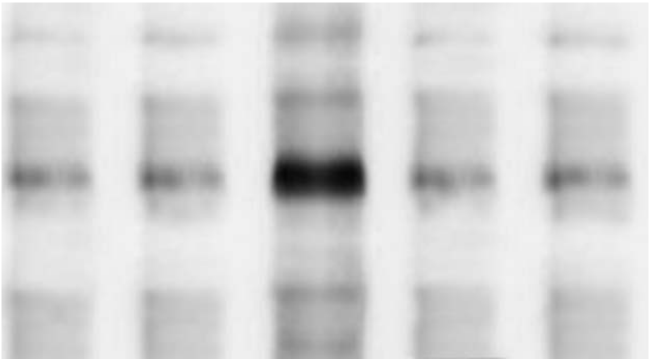

Fig.4B. pSTAT3

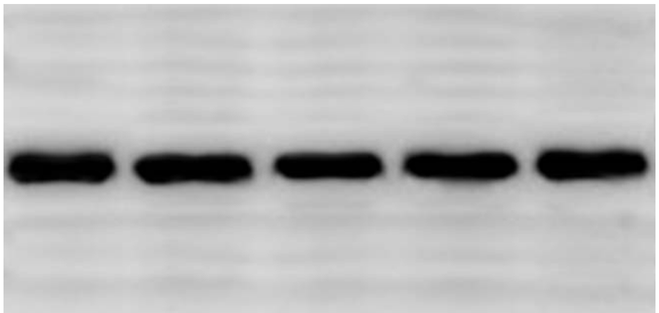

Fig.4B. Actin

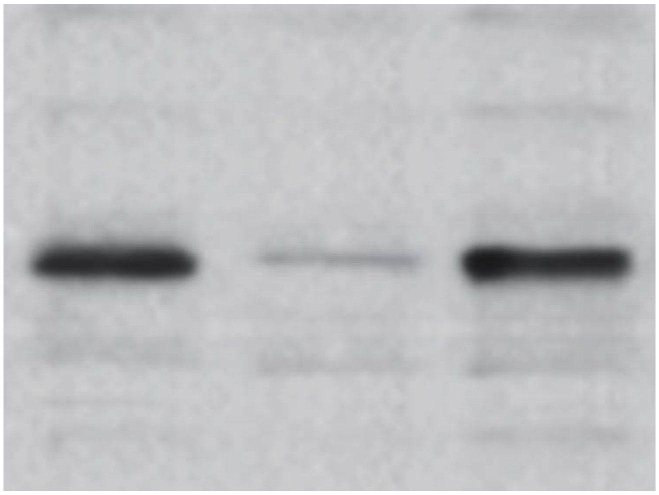

Fig.4C. p300

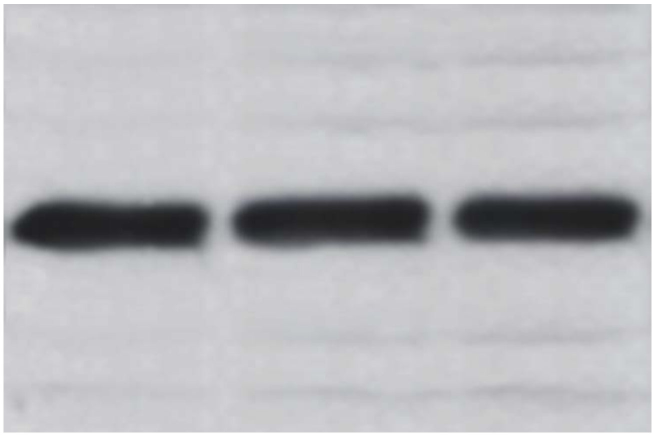

Fig.4C. Actin

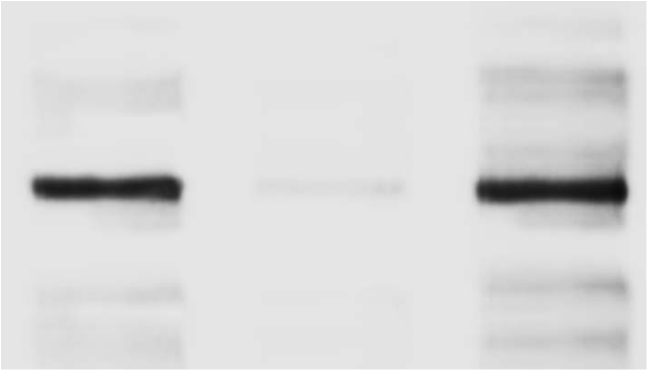
Fig.4D. STAT3

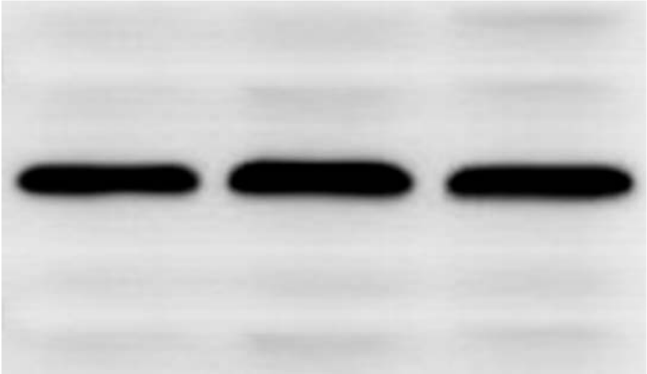
Fig.4D. Actin

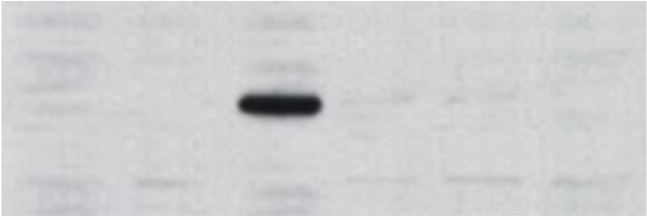
Fig.4G. IL-10

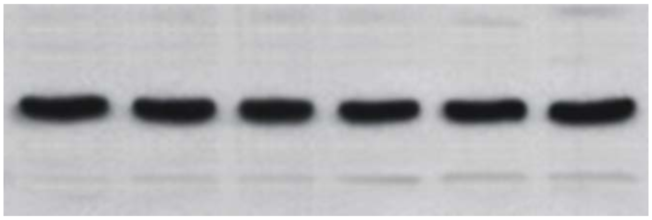
Fig.4G. Actin
